# Keratinocyte-Immune Cell Crosstalk in a STAT1-Mediated Pathway: Novel Insights Into Rosacea Pathogenesis

**DOI:** 10.3389/fimmu.2021.674871

**Published:** 2021-07-05

**Authors:** Zhili Deng, Fangfen Liu, Mengting Chen, Chuchu Huang, Wenqin Xiao, Sini Gao, Dan Jian, Yuyan Ouyang, San Xu, Jinmao Li, Qian Shi, Hongfu Xie, Guohong Zhang, Ji Li

**Affiliations:** ^1^ Department of Dermatology, Xiangya Hospital, Central South University, Changsha, China; ^2^ Hunan Key Laboratory of Aging Biology, Xiangya Hospital, Central South University, Changsha, China; ^3^ Key Laboratory of Organ Injury, Aging and Regenerative Medicine of Hunan Province, Central South University, Changsha, China; ^4^ National Clinical Research Center for Geriatric Disorders, Xiangya Hospital, Central South University, Changsha, China; ^5^ Key Laboratory of Molecular Radiation Oncology Hunan Province, Changsha, China; ^6^ Department of Pathology, Shantou University Medical College, Shantou, China; ^7^ Department of Dermatology, The Second Affiliated Hospital of Xinjiang Medical University, Urumqi, China

**Keywords:** rosacea, RNA-Seq, STAT1, skin inflammation, keratinocyte, pathogenesis 2, epithelial-immune cell crosstalk

## Abstract

Rosacea is a common chronic inflammatory condition that mainly affects the central face. However, the molecular background of the normal central face and the transcriptional profiling and immune cell composition of rosacea lesions remain largely unknown. Here, we performed whole-skin and epidermal RNA-seq of central facial skin from healthy individuals, lesions and matched normal skin from rosacea patients. From whole-skin RNA-seq, the site-specific gene signatures for central facial skin were mainly enriched in epithelial cell differentiation, with upregulation of the activator protein-1 (AP1) transcription factor (TF). We identified the common upregulated inflammatory signatures and diminished keratinization signature for rosacea lesions. Gene ontology, pathway, TF enrichment and immunohistochemistry results suggested that *STAT1* was the potential core of the critical TF networks connecting the epithelial–immune crosstalk in rosacea lesions. Epidermal RNA-seq and immunohistochemistry analysis further validated the epithelial-derived *STAT1* signature in rosacea lesions. The epidermal STAT1/IRF1 signature was observed across ETR, PPR, and PhR subtypes. Immune cell composition revealed that macrophages were common in all 3 subtypes. Finally, we described subtype-specific gene signatures and immune cell composition correlated with phenotypes. These findings reveal the specific epithelial differentiation in normal central facial skin, and epithelial–immune crosstalk in lesions providing insight into an initial keratinocyte pattern in the pathogenesis of rosacea.

## Introduction

Rosacea is a common disorder of the facial skin that affects about 5.5% of individuals in the general population (1). It is divided into four subtypes: erythematotelangiectatic (ETR), papulopustular (PPR), phymatous (PHR) and ocular. Convex parts of the central face are the region of the skin most frequently affected by rosacea. In fact, human skin shows remarkable diversity in its structure and function across anatomic sites (2). The embryologic development of the facial dermis derives from the neural crest, so facial skin has a distinct developmental origin different from other anatomic sites (3). Recently, a new concept of the immunological anatomy of skin highlighted the epithelial–immune interaction of skin in different anatomic sites (4). However, the molecular characteristics of the central facial skin and their potential relation with the pathogenesis of rosacea remain unknown.

Although the pathogenesis of rosacea is not fully understood, it is defined as a chronic, inflammatory skin condition. Abnormalities in the innate immune response system and neurovascular dysregulation are considered primarily implicated in the pathophysiology of rosacea (5). Immune cells involved in the innate immune response, such as macrophages, mast cells, and neutrophils, were described in rosacea lesions (6). However, the immune cell composition as well as the relative change in abundance of specific cell types in rosacea lesions are still largely undefined.

Dynamic epithelial–immune crosstalk fine-tunes epithelial homeostasis (7), which suggests that epithelial cells (keratinocytes) might be the primary trigger of the innate immune response at the onset and development of rosacea. However, evidence of keratinocyte–immune cell crosstalk in the rosacea lesion and what initially triggers this crosstalk remain poor unknown. Dynamic rosacea appears to feature a consistent inflammatory continuum, and a ‘‘developmental march’’ among different subtypes was assumed (8). Regardless, little is known about the distinct inflammatory properties of different clinical subtypes and their relationship with clinical manifestations.

To address these gaps in our understanding, the transcriptional profiling and the immune cell composition in rosacea, we used RNA-sequencing (RNA-seq) of central facial skin from healthy individuals and a large number of central facial skin lesions and matched normal skin surrounding the auricle from patients with different rosacea subtypes.

## Materials and Methods

### Sample Collection

Skin biopsies (within 0.5 cm^2^) were obtained from the central facial skin of female healthy individuals undergoing plastic surgery (n=19 for whole skin RNA extraction; n=5 for epidermis RNA extraction; epidermis was isolated from whole skin with dispase) and lesions of the central face and corresponding normal skin surrounding the auricle from female patients with ETR (n=15 for whole skin; n=6 for epidermis), PPR (n=22 for whole skin; n=8 for epidermis) and PHR (n=9 for whole skin; n=4 for epidermis) (aged 20–60 years) from the Department of Dermatology in Xiangya Hospital, Central South University. The study was approved by the ethics committee of Xiangya Hospital, Central South University, and written informed consent was obtained from all participants. The experiments conformed to the principles set out in the WMA Declaration of Helsinki and the Department of Health and Human Services Belmont Report.

### Library Construction and Sequencing

Whole skin or epidermis RNA was isolated by using TRIzol Reagent (Thermo Fisher Scientific, USA). A total of 3 µg RNA per sample was used as input material for RNA sample preparations. Sequencing libraries were generated by using the NEBNext UltraTM RNA Library Prep Kit for Illumina (NEB, USA) following the manufacturer’s recommendations. The Illumina PE150 libraries were sequenced on a Hiseq 4000 platform according to the manufacturer’s recommendations (Illumina) at the Novogene Bioinformatics Institute, Beijing. Finally, 150-bp paired-end reads were generated.

### Quantification of Gene Expression

Raw data (raw reads) in the FASTQ format were first processed through in-house Perl scripts. In this step, clean data (clean reads) were obtained by removing reads containing adapters and ploy-N and low-quality reads from raw data. Raw reads were aligned to the human reference genome (hg19). Sequencing data were checked for sequencing quality by using FASTQC. FeatureCounts v1.5.0-p3 was used to count the read numbers mapped to each gene. Gene expression was estimated by using fragments per kilobase transcript sequence per million base pairs sequenced (FPKM) values.

### Differential Gene Expression Identification

Differentially expressed genes (DEGs) were identified by using the DESeq2 R package (1.16.1). P-values were adjusted by the Benjamini and Hochberg’s approach to control the false discovery rate (FDR). Then, genes with FDR value < 0.05 with fold-change in expression >2 on DESeq2 were considered differentially expressed. Volcano plots were created by using ggplot2 v2.2.1 in R to visualize the top DEGs. Hierarchical clustering with Pearson’s correlation of DEGs involved using R software. Venn diagrams were created with the online software Venny (http://bioinfogp.cnb.csic.es/tools/venny).

### Gene Ontology (GO) and Kyoto Encyclopedia of Genes and Genomes (KEGG) Pathway Analyses

We performed gene set enrichment analysis (GSEA) to identify GO terms enriched in DEGs (9). Furthermore, statistically enriched biological processes or pathways of DEGs were ranked and classified by using the Database for Annotation, Visualization and Integrated Discovery (DAVID v6.7) for GO and KEGG pathways (http://david.abcc.ncifcrf.gov/home.jsp), and network analysis involved using Metascape (http://metascape.org).

### Integrated Analyses of Transcription Factors (TFs)

Human TFs were obtained from the official list of human TFs from Lambert et al. (http://humantfs.ccbr.utoronto.ca/download.php) (10) and human transcription database (http://bioinfo.life.hust.edu.cn/HumanTFDB#!/). Transcriptional regulatory interactions were evaluated in the TF-target interaction database for humans, TRRUST v2 (https://www.grnpedia.org/trrust/). Upregulated TFs were subjected to enrichment analysis by using ChIP-X Enrichment Analysis v3 (ChEA3, https://amp.pharm.mssm.edu/ChEA3) (11). TFs and their interconnected autoregulatory loops were obtained from database of Core transcriptional Regulatory Circuitries (dbCoRC, http://dbcorc.cam-su.org/) (12).

### Assessment of Immune Cell Types

Relative levels of distinct immune cell types in rosacea lesions were quantified by using the xCell algorithm (13) and CIBERSORT algorithm (https://cibersort.stanford.edu) (14).

### Immunohistochemistry

Immunohistochemistry staining for STAT1, IRF1, IRF8 was conducted as described previously (15). Briefly, skin sections were incubated with antibodies: Rabbit anti-phospho-c-Jun (1:200, Cell signaling, catalog 3270), Rabbit anti-FosB (1:50, Cell signaling, catalog 2251), Rabbit anti-phospho-STAT1 (1: 800, Cell signaling, catalog 9167), Rabbit anti-IRF1 (1:400, Cell signaling, catalog 8478), Rabbit anti-IRF8 (1:2000, Abcam, catalog ab207418). As negative controls, Rabbit IgG isotype control (1: 400, Cell signaling, catalog 3900) was used (data shown in **Supplementary Figure 5**). Images were obtained from three typical areas for each sample. The quantification of nuclear localization of each protein was evaluated with ImageJ.

### Statistical Analysis

Statistical analysis was conducted with SPSS 20.0 and GraphPad 8.0. Data are presented as the mean ± SEM. We evaluated the data for normal distribution and similar variance between groups. Statistical significance (*P < 0.05, **P < 0.01) was assessed by 2-tailed unpaired Student’s t-test for comparisons between 2 groups and 1-way analysis of variance (ANOVA) with relevant *post hoc* tests for multiple comparisons. We performed the 2-tailed Mann-Whitney U test for statistical analysis when the data were not normally distributed or exhibited unequal variances between the two groups.

## Results

### Normal Central Facial Skin Presents Anatomy-Specific Gene Signatures Involved in Epithelial Cell Differentiation

**Figure 1:**
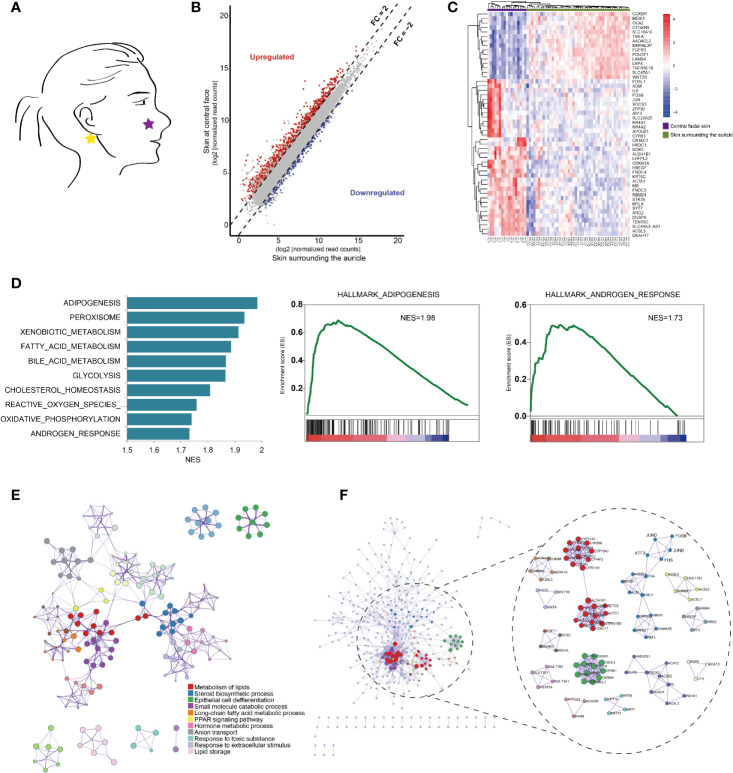
Anatomical transcriptional profiling in central facial skins. **(A)** Anatomic distribution of representative skin samples of central face tissue from healthy volunteers (N=17) and normal skin surrounding the auricle in rosacea patients (N=45). **(B)** Scatter plots comparing the expression of genes with fold change in expression > 2, false discovery rate (FDR) < 0.05 in central facial skin (purple bar) and normal skin surrounding the auricle (green bar). **(C)** Heatmap of the top 50 differentially expressed genes (DEGs) between central facial skin and skin surrounding the auricle. **(D)** Gene set enrichment analysis (GSEA) highlighting genes involved in adipogenesis, androgen and estrogen responses in central facial skin. NES, normalized enrichment score. **(E)** Metascape network analysis of enriched gene ontology (GO) terms and KEGG pathways for all DEGs, indicating crosstalk between the metabolism of lipid and steroid biosynthetic process in central facial skin. **(F)** The complex interactome network generated by Metascape, with the complexes colored according to identities. Highlights the expanded visualization of the complex and the AP1 pathway, including FOS, FOSB, JUNB, JUND and ATF3.

In an initial analysis, we used transcription profiling of central facial skin from healthy individuals (healthy skin [HS], n=17) compared to normal skin surrounding the auricle (normal skin [NS], n=45) (**Figure 1A**). We identified 984 differentially expressed genes (DEGs) (fold change in expression > 2 and false discovery rate [FDR] < 0.05): 799 upregulated and 185 downregulated in central facial skin (**Figure 1B**). Hierarchical clustering analysis distinguished HS from NS, thus indicating a different gene expression pattern between the two anatomic sites (**Figure 1C**).

To further characterize the gene signatures, we used gene set enrichment analysis (GSEA) to identify hallmark gene sets enriched among the DEGs of HS. Genes associated with adipogenesis and androgen responses were enriched among those upregulated in HS (**Figure 1D**). As expected, the top clusters with their representative terms were enriched biological processes, including metabolism of lipids (Log10(q)= -38.12), steroid biosynthetic process (Log10(q)= -19.77) and epithelial cell differentiation (Log10(q)= -14.74) (**Figure 1E**). Furthermore, the molecular complex detection (MCODE) algorithm subclustered protein–protein interaction (PPI) networks into 13 subclusters, including steroid biosynthetic process (Log10(p)= -12.5) and cornification (Log10(p)= -9.4) (**Figure 1F**).

**Figure 2:**
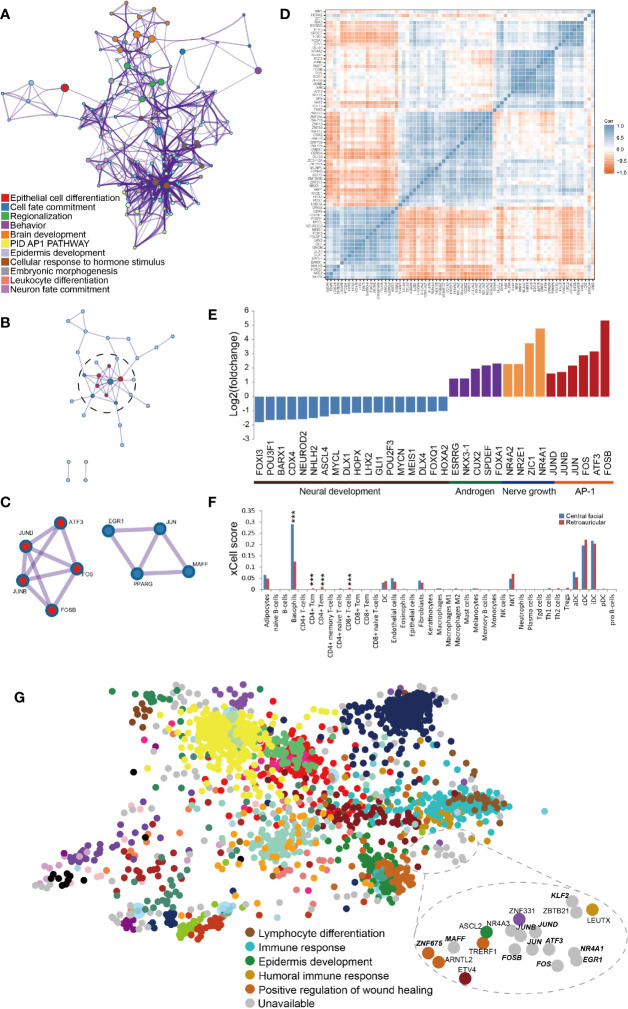
Transcription factors (TFs) related to transcriptional profiling in central facial skin. **(A)** Metascape network analysis of enriched GO terms and KEGG pathways for TFs highlights cellular response to hormone stimulus, brain development and regionalization biological processes. **(B)** Metascape network analysis of the complex interactome network for TFs indicating the connection between the AP1 pathway and cellular response to hormone stimulus. **(C)** Genes composing the complex of the AP1 pathway (red color) and cellular response to hormone stimulus (blue color). **(D)** Correlation heatmap representing significant correlation of expression of TFs by transcripts per million kilobases (TPM). Blue squares indicate significant positive correlation (r > 0.5, p < 0.05), white squares non-significant correlation (p > 0.05), and red squares significant negative correlation (r < −0.5, p < 0.05). **(E)** Manual classification of biological process for TFs by their function. **(F)** The xCell-inferred enrichment score of cell types in central facial skin (blue color) and skin surrounding the auricle (red color). The xCell scores predict relative enrichment for cell types, not the extract proportions. ***P<0.001. **(G)** TF cluster generated by ChEA3, which prioritizes TFs based on overlap between our TFs and previously annotated TF targets assembled from multiple resources. The TFs identified in this study are highlighted by bold italics. The results demonstrate TFs enriched in the AP1 pathway and cellular response to hormone stimulus.

To examine the transcriptional mechanisms underlying the DEGs, we analysed transcription factors (TFs). A total of 74 TFs were identified, including 21 downregulated and 53 upregulated, in HS. The top clusters and networks involved the biological processes epithelial cell differentiation (Log10(q)= -6.81), cell fate commitment (Log10(q)= -6.74) and regionalization (Log10(q)= -6.74) (**Figure 2A**). The MCODE algorithm subclustered the PPI network into 2 subclusters: the activator protein-1 (AP1) pathway (Log10(p)= -11.5) and cellular response to hormone stimulus (Log10(p)= -9.2) (**Figures 2B, C**). The co-expressed TFs had the most significantly enriched functional annotations (**Figure 2D**). We developed a scheme to classify TFs in terms of their biological function: downregulated TFs were mostly clustered in neural development and upregulated TFs in nerve growth and the AP1 pathway (**Figure 2E**). Furthermore, we identified AP1 TFs that were covered in the ChEA3 database, including *JUN*, *JUND*, *JUNB*, *FOS*, *FOSB*, A*TF3* and *MAF*F (**Figure 2G**). By immunohistochemistry, we further confirmed that AP1 pathway was significantly upregulated in the central facial skin (**Supplementary Figure S1**). Thus, gene signatures involved in metabolism of lipids and epithelial cell differentiation (cornification) were characteristics of central facial skin, and AP1 TFs may be required for maintaining homeostasis in HS.

To accurately and comprehensively estimate the cell types of HS, we used absolute deconvolution with the xCell algorithm. As compared with skin surrounding the auricle, in HS, the number of basophils and CD4^+^ and CD8^+^ T cells was significantly increased (**Figure 2F**), which indicates an inflammation-sensitive background.

### Transcriptional Profiling Highlighted Gene Signatures Involved in the Immune Response and Epithelial Cell Differentiation in Rosacea Lesions

To further characterize the gene signatures in more detail, we used differential analysis with comparison. First, we compared rosacea lesions (LS, n=42) and normal central facial skin (n=17) from healthy individuals (LS *vs* HS). With 584 genes downregulated and 386 upregulated, rosacea lesions had distinct gene expression profiles (**Supplementary Figures 2A, B**). Downregulated genes showed enrichment of biological processes related to cellular response to hormone stimulus (Log10(q)= -5.65). Molecular function analysis revealed enriched connective tissue development (Log10(q)= -4.92) and epithelial cell differentiation (Log10(q)= -4.87) in rosacea lesions (**Supplementary Figure S2C**). However, the top GO terms for upregulated genes were mainly related to immune response, including regulation of innate immune response (Log10(q)= -25.16), defense response to other organism (Log10(q)= -24.94) and response to bacterium (Log10(q)= -20.69) (**Supplementary Figure S2D**). We identified 55 downregulated and 17 upregulated TFs in rosacea lesions (**Supplementary Figure S2E**). Metascape GO terms were mesenchyme development (Log10(q)= -9.76), epithelial cell differentiation (Log10(q)= -5.17), and temperature homeostasis (Log10(q)= -2.61) (**Supplementary Figure S2F**). By classifying TFs in terms of expression, the scheme showed the AP1 TFs (*FOS*, *FOSB*, *JUN* and *JUND*) downregulated in rosacea lesions (**Supplementary Figure S2G**).

To ensure the transcriptional profiling in rosacea lesions, we then compared rosacea lesions with matched normal skin surrounding the auricle from the same patients (LS *vs* NS; n=42). In total, 1040 genes were upregulated and 606 downregulated (**Supplementary Figures S3A, B**). The main GO terms for downregulated DEGs were the biological processes skin development (Log10(q)= -21.15), cell morphogenesis involved in differentiation (Log10(q)= -9.41) and chemical synaptic transmission (Log10(q)= -4.33) (**Supplementary Figure S3C**). The top upregulated GO terms were mainly related to the immune response, including response to bacterium (Log10(q)= -47.55), lymphocyte activation (Log10(q)= -44.93) and adaptive immune response (Log10(q)= -42.78) (**Supplementary Figure S3D**). Overall, 94 differentially expressed TFs were identified, mainly involved in the pattern specification process (**Supplementary Figures S3E–G**).

**Figure 3:**
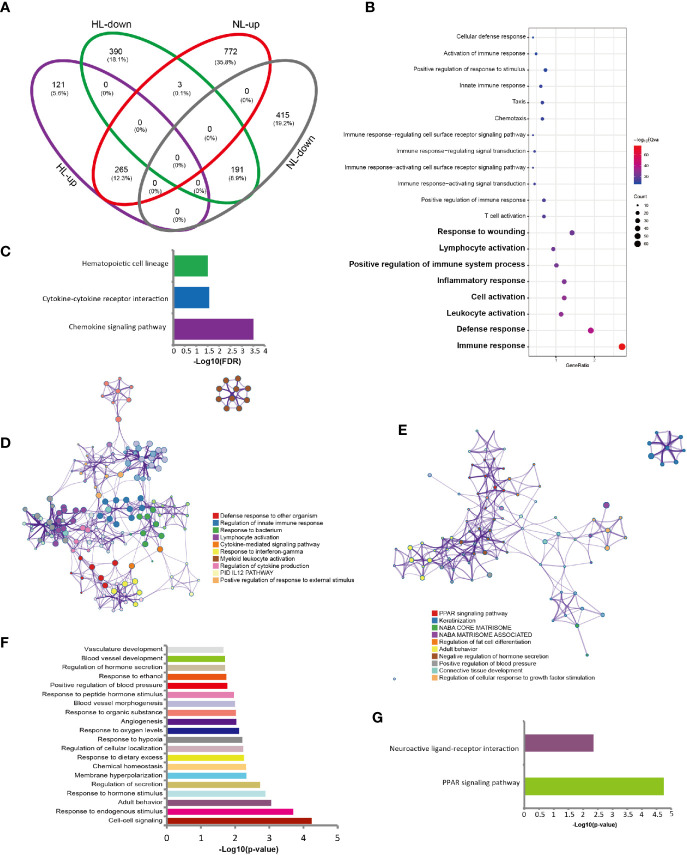
Gene signatures in rosacea lesions compared with central facial skin and skin surrounding the auricle. **(A)** Venn plot of the DEGs shared between rosacea lesions and matched normal skin surrounding the auricle (LS *vs* NS) and central facial skin from healthy controls (LS *vs* HS). HL: LS *vs* HS; NL: LS *vs* NS; up: upregulated genes; down: downregulated genes. LS, lesion skin; HS, healthy skin; NS, matched normal skin. **(B)** Go terms for the 265 shared upregulated genes showing the immune-related biological processes. **(C)** Results of KEGG pathway enrichment analysis of 265 shared upregulated genes. **(D)** Metascape network analysis of the GO terms enriched for the 265 shared upregulated genes involved in immune responses, including defense response to other organism, and regulation of innate immune response. **(E)** Metascape network analysis of the enrichment of the 191 shared downregulated genes for biological processes, such as the PPAR signaling pathway, and keratinization. **(F)** GO terms for the 191 shared downregulated genes by DAVID analysis. **(G)** Enriched pathways for the 191 shared downregulated genes, showing PPAR signaling pathway.

Finally, we observed a gene set overlapping between LS versus HS and LS versus NS: 456 genes overlapped; 191 were downregulated and 265 upregulated (**Figure 3A**). We investigated whether the commonly expressed genes indicated a change in biological process. For the upregulated genes, the enriched GO terms included the immune response (FDR= 3.36E-33), defense response (FDR=2.65E-17) and response to wounding (FDR=5.09E-10) (**Figure 3B**). Total 39 genes were enriched in the GO term of immune response. The interferon-inducible chemokines CXCL9 and CXCL10 ranked on the top of this gene list (**Supplementary Table S1** and **Supplementary Figure S4**). Macrophages and activated keratinocytes were the main producers of CXCL9 and CXCL10 (16). KEGG pathway analysis highlighted the chemokine signaling pathway (FDR=3.14 E-4) (**Figure 3C**). The immune-related biological processes were validated, including defense response to other organisms (Log10(q)= -23.11), regulation of innate immune response (Log10(q)= -21.36), response to bacterium (Log10(q)= -20.64) and response to interferon-γ (IFN-γ) (Log10(q)= -20.64) (**Figure 3D**).

Regarding downregulated genes, Metascape network analysis revealed the PPAR signaling pathway (Log10(q)= -1.55) and keratinization (Log10(q)= -1.17) (**Figure 3E**). GO analysis with DAVID did not reveal significant biological processes. Therefore, we show the *P* values for GO terms in **Figure 3F**. KEGG pathway analysis for downregulated genes highlighted the PPAR signaling pathway (FDR=0.02) (**Figure 3G**).

We reveal a common gene signature derived from upregulated genes, enriched in biological processes including inflammation, and downregulated genes, involved in PPAR signalling and keratinization.

### Signal Transducer and Activator of Transcription 1 (STAT1)-Related Regulatory Loop Responding to Inflammation in Rosacea Lesions

**Figure 4:**
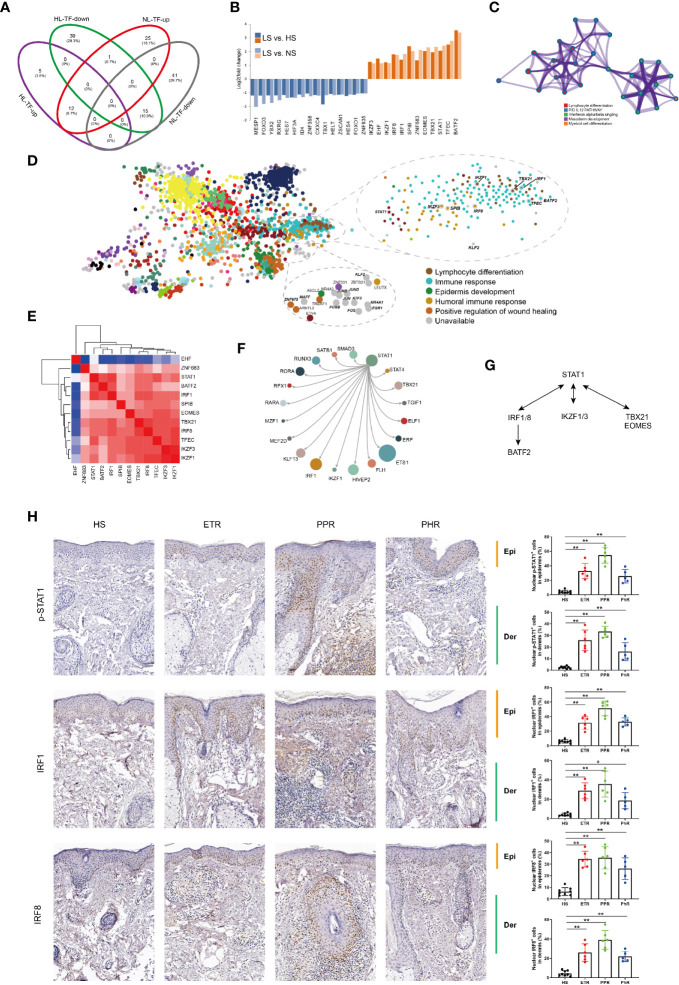
STAT1-mediated inflammatory signatures for rosacea lesions. **(A)** Venn diagram showing the overlap genes between rosacea lesions and normal skin surrounding the auricle and central facial skin. HL: LS *vs* HS; NL: LS *vs* NS; up: upregulated genes; down: downregulated genes. LS, lesion skin; HS, healthy skin; NS, matched normal skin; TF, transcription factor. **(B)** Fold change for the TFs shared between PL *vs* HN and PL *vs* PN comparisons. **(C)** Metascape network analysis of TFs with the GO term of immune responses. **(D)** Enrichment of TFs generated by the ChEA3 database, and the 9 genes covered are marked, including STAT1, lymphocyte differentiation (IKZF1, TBX21, IRF1), immune response (TFEC, BATF2) and humoral immune response (IKZF3, SPIB, IRF8). **(E)** Correlation heatmap of upregulated TFs. Red color indicates correlation. **(F)** The core regulatory circuitry of the TF STAT1 generated by the dbCoRC database (a database of core transcriptional regulatory circuitries), showing the interaction of STAT1 and TBX21, IKZF1and IRF1. **(G)**. The hypothesized scheme of target genes of STAT1 in different immune cells. **(H)** Immunohistochemistry staining for validation of p-STAT1, IRF1 and IRF8 expression. HS, healthy skin (n = 8); ETR, erythematotelangiectatic (n = 6); PPR, papulopustular (n = 6); PHR, phymatous (n = 5). Right panel, the quantification of nuclear localization of p-STAT1, IRF1 and IRF8 in epidermis and dermal infiltration of rosacea lesions. Data represent the mean ± SEM. *P < 0.01, **P < 0.01. 1-way ANOVA with Bonferroni’s *post hoc* test was used.

We found 27 overlapped TFs differentially expressed in rosacea lesions by comparing LS versus HS and LS versus NS: 15 were downregulated and 12 upregulated (**Figures 4A, B**). Metascape network analysis identified lymphocyte differentiation (Log10(q)= -4.52), interleukin 12 (IL12) (Log10(q)= -2.03) and IFN alpha/beta (Log10(q)= -2.01) signaling pathways (**Figure 4C**). Summary of enrichment analysis in TRRUST indicated TFs potentially regulated by STAT1 (Log10(p)= -4.00).

Furthermore, the ChEA3 database covered 9 of 11 TFs identified, including STAT1, lymphocyte differentiation genes (*IKZF1*, *TBX21* and *IRF1*), immune response genes (*TFEC* and *BATF2*) and humoral immune response genes (*IKZF3*, *SPIB* and *IRF8*) (**Figures 4D, E**). Given that the core transcription regulatory circuitry (CRC) consists of a small group of self-regulated TFs and their interconnected regulatory loops, dbCoRC analysis showed a STAT1-containing core regulatory circuitry, including the interactions STAT1–interferon regulatory factor 1 (IRF1) and STAT1–IRF8 (**Figure 4F**). We manually analysed the correlation between TFs and constructed the regulatory network (**Figure 4G**). Consistently, by immunohistochemistry we verified the increased nuclear localization of phosphor-STAT1, IRF1 and IRF8 in both epidermis and dermal infiltration of rosacea lesions (**Figure 4H**).

### Epidermal RNA-Seq Data Further Revealed an Epithelial-Derived STAT1 Signature in Rosacea Lesions

**Figure 5:**
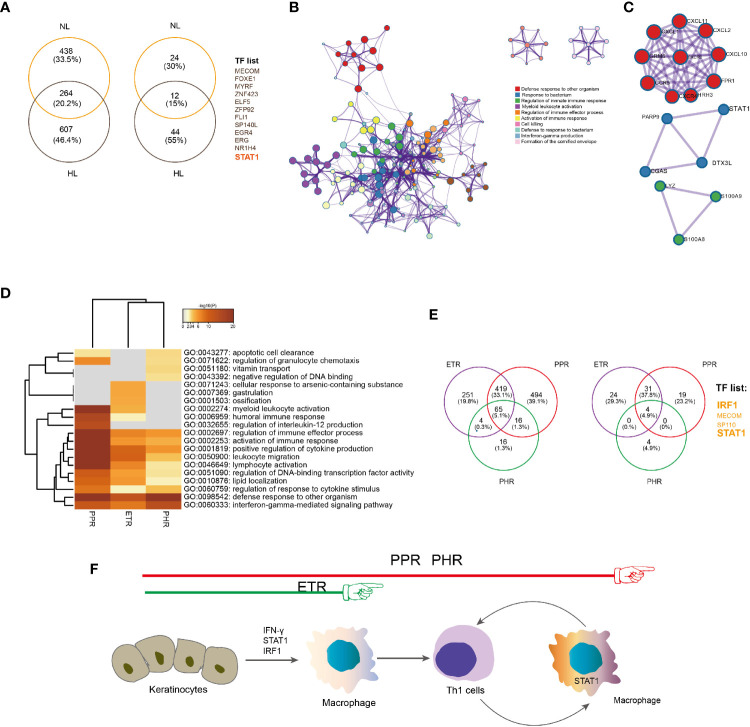
Epidermal-derived IFNγ/STAT1/IRF-1 signature in rosacea lesions. **(A)** Venn diagram showing the overlap between upregulated genes (left) and TFs (right) in rosacea lesions and matched normal skin surrounding the auricle (LS *vs* NS) and central facial skin from healthy controls (LS *vs* HS). HL: LS *vs* HS; NL: LS *vs* NS. **(B)** Metascape network analysis of the enrichment of the 264 shared upregulated genes related to the biological process IFNγ production. **(C)** Protein–protein interaction constructed by the 264 shared genes indicate the *STAT1*, *PARP9*, *DTX3L* and *CGAS* interaction. **(D)** Metascape GO-term heatmap showing the IFNγ signaling pathway shared across ETR, PPR and PHR subtypes. **(E)** Venn diagram showing the overlap between upregulated genes (left) and transcription factors (right) in ETR, PPR and PHR subtypes compared with central facial skin from healthy controls. **(F)** Proposed model for IFNγ/STAT1/IRF-1 signature triggering activation of inflammatory responses in rosacea lesions. Hypothesized immune cells involved in rosacea from ETR to PPR and PHR.

To confirm whether the *STAT1* signature derived from epidermis contributed to rosacea lesions, we used epidermal RNA-seq and identified a gene set overlapping between LS (n=18) versus HS (n=5) and LS (n=18) versus NS (n=18), with 264 genes showing elevated expression in both comparisons. For the upregulated genes, the enriched GO terms included defense response to other organism (Log10(q)= -23.12) and response to bacterium (Log10(q)= -17.02). Among the 264 upregulated genes were 12 overlapped TFs in rosacea lesions, including STAT1, FOXE1 and ERG (**Figure 5A**). These data confirmed that the epithelial-derived STAT1 signature contributed to rosacea lesions.

### Epidermal IFNγ/STAT1/IRF-1 Shared Across ETR, PPR and PHR Subtypes

To identify the common epidermal gene signatures across rosacea subtypes, we compared ETR (n= 6), PPR (n= 8), and PHR (n=4) subtypes with normal central facial skin from healthy individuals (HS, n=5). Enrichment analysis of upregulated genes revealed an IFN-γ-mediated signaling pathway across ETR, PPR and PHR subtypes (**Figures 5B–D**). We further observed the common 65 upregulated genes shared across the 3 subtypes: 4 TFs were identified, namely STAT1, IRF1, MECOM and SP110 (**Figure 5E**). By immunohistochemistry we also demonstrated that the nuclear localization of phosphor-STAT1 and IRF1 was increased in epidermal cells of rosacea lesions (**Figure 4H**). These results demonstrated the STAT1/IRF1 signature across the ETR, PPR and PHR subtypes.

### Subtype-Specific Gene Signature

**Figure 6:**
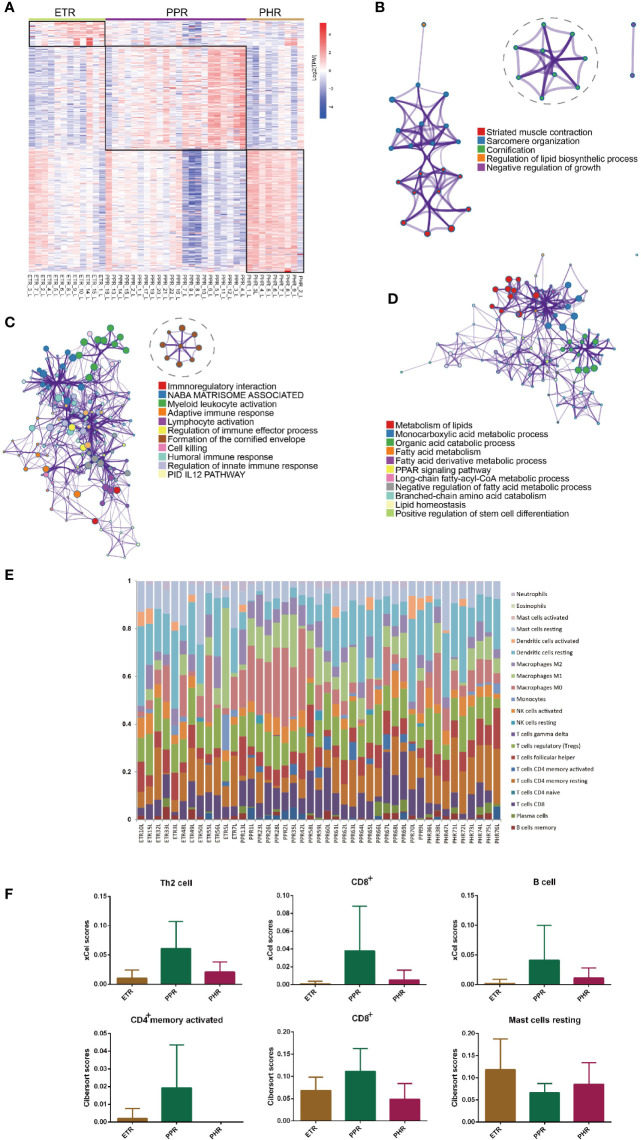
Subtype-specific gene signatures and immune cells across ETR, PPR and PHR subtypes. **(A)** Heatmap of the different upregulated genes among the subtypes. **(B)** Metascape network analysis of enrichment for upregulated genes in ETR subtype. **(C)** Metascape network analysis of enrichment for upregulated genes in PPR subtype. **(D)** Metascape network analysis of enrichment for upregulated genes in PHR subtype. **(E)** The composition of 22 immune cells for each sample across ETR, PPR and PHR subtypes by CIBERSORT algorithm. **(F)** Difference in number of immune cells among ETR, PPR and PHR subtypes. The xCell algorithm identified immune cells including T helper 2 cells, CD8^+^ T cells and B cells, and the CIBERSORT algorithm revealed the different abundance of resting mast cells, and activated CD4^+^ memory and CD8^+^ cells among ETR, PPR and PHR subtypes.

To elucidate the gene signature differences among ETR, PPR and PHR subtypes, we compared one subtype with the other two subtypes. The heatmap (**Figure 6A**) shows that each subtype has a unique expression pattern. To determine whether these subtypes are specific to biological processes, we examined upregulated gene enrichment. As compared with PPR and PHR subtypes, ETR showed strong enrichment for biological processes related to muscle contraction (Log10(q)= -4.63) and cornification (Log10(q)= - 1.53). As compared with ETR and PHR subtypes (**Figure 6B**), PPR showed a strong enrichment of both innate (Log10(q)= -11.72) and adaptive immune responses (Log10(q)= -7.55) (**Figure 6C**). We also noted a strong enrichment of formation of cornified envelope (Log10(q)= -8.99) in the PPR subtype. Of note, PHR was mainly related to metabolism of lipids (Log10(q)= -18.32), monocarboxylic acid metabolic process (Log10(q)= -15.49) and organ acid catabolic process (Log10(q)= -12.10) (**Figure 6D**).

### Immune Cell Composition for ETR, PPR and PHR Subtypes

Using the CIBERSORT algorithm, we first investigated the common and different immune infiltration features among ETR, PPR and PHR subtypes (**Figure 6E**). Resting dendritic cells and macrophages were common to all 3 subtypes. In the ETR subtype, the most abundant immune cells were resting dendritic cells (18.3%), regulatory T cells (12.6%) and resting mast cells (11.9%), whereas in the PPR subtype, the most abundant immune cells were M0 macrophages (13.9%), resting dendritic cells (12.7%) and regulatory T cells (11.9%). The top 3 abundant immune cell types in the PHR subtype were resting dendritic cells (18.8%), resting CD4^+^ memory T cells and regulatory T cells (11.1%). As compared with PPR and PHR subtypes, ETR showed a higher proportion of resting mast cells, whereas as compared with ETR and PHR subtypes, PPR showed abundant activated memory CD4^+^ and CD8^+^ T cells by xCell and CIBERSORT algorithms (**Figure 6F**).

## Discussion

Previous whole-skin transcriptome analysis revealed that Th1/Th17 polarized inflammation and macrophage infiltration were hallmarks in ETR, PPR and PHR subtypes (6), and separated signals of immune and keratinization gene expression in PPR (17). In addition, prominent permeability barrier alterations had also been descried in PPR *via* whole-skin RNA sequencing analysis (18). In the present study, through both whole-skin and epidermal transcriptome analysis, we characterized the atlas of transcriptomic profiles indicating the site-specific gene signatures and epidermal homeostasis for central facial skin. We further identified common inflammatory signatures, the TF network in whole-skin and epidermis, and epidermal STAT1/IRF1 signature and epithelial–immune crosstalk for rosacea lesions (**Figure 5F**). We also compared the molecular characteristics and immune cell composition for different rosacea subtypes based on their transcriptome profiles.

### Normal Central Facial Skin Exhibited Anatomy-Specific Molecular Characteristics Involved in Metabolism of Lipids, Steroid Biosynthetic Process and Epithelial Cell Differentiation

We compared transcription profiling of central facial skin from healthy individuals and normal skin surrounding the auricle. Upregulated gene signatures were mainly associated with metabolism of lipids, steroid biosynthetic process and epithelial cell differentiation.

We identified enrichment of the epithelial-cell differentiation pathway and AP1 TFs (*FOS*, *FOSB*, *JUN* and *JUN*) in central facial skin. AP1 TFs are key regulators of epidermal keratinocyte survival and differentiation (19). The epidermis contains proliferating keratinocytes that differentiate to form the corneal layer. Corneocytes together with intercellular lipids isolate the body from the external environment and protect it against infection. Thus, persistent activation of AP1 pathways may be necessary to maintain keratinocyte differentiation and the structural barrier of central facial skin, and its dysfunction might lead to rosacea. Our results were consistent with the hypothesis that AP1 TFs (*FOS*, *FOSB*, *JUN* and *JUND*) were downregulated in rosacea lesions. This situation may reflect intrinsic defects accelerating keratinocyte differentiation capacity, which may contribute to compromised barrier function in rosacea lesions.

To summarize, the DEGs associated with keratinocyte differentiation are required for maintenance of the local homeostasis of central facial skin and might explain the regional difference in rosacea susceptibility.

### STAT1 Mediated Inflammatory Signatures for Rosacea Lesions

Although the clinical manifestations of rosacea are heterogeneous, they are all related to the presence of characteristic facial or ocular inflammation involving both the vascular and tissue stroma (20). We attempted to present a common link between the triggers and the immune response of rosacea lesions and used a comprehensive comparison among central facial skin, skin surrounding the auricle and rosacea lesions to identify shared gene signatures for rosacea lesions (**Figure 5F**).

The common upregulated gene signatures of rosacea lesions were mainly associated with innate immune responses including the defense response to other organisms and response to IFN-γ, which promotes macrophage activation, mediates antiviral and antibacterial immunity, enhances antigen presentation, and orchestrates activation of the innate immune system. Consistently, we identified 8 of 12 TFs (*STAT1*, *IRF1*, *IRF8*, *IKZF1*, *IKZF3*, *TBX21*, *EOMES* and *BATF2*) involved in IFN-γ signaling. We also confirmed the interaction of the 8 TFs by co-expression and functional enrichment. Finally, core TF loop analysis revealed that STAT1 is the core TF of the IFN-γ/STAT1 pathway.

Familial rosacea associated with a gain-of-function mutation (C324R) in STAT1 suggests that STAT1 could lead to chronic facial inflammation as the cause of rosacea (21). Innate immune-system perturbations are known to contribute to the pathogenesis of rosacea (20). The IFNγ/STAT1/IRF-1 signaling pathway was previously found involved in the processes of keratinocyte differentiation and the inflammatory interplay between innate immune responses and keratinocytes (22). The macrophage IRF8/IRF1 regulome was found required for protection against infection and is associated with chronic inflammation (23). IKZF1 regulates dendritic cell development and cytokine signaling pathways and CD4^+^ Th cell differentiation (24). TBX21 and EOMES are the only T-box proteins expressed in the immune system and control key checkpoints of natural killer cell maturation (25). We also identified the up-regulated transcription factors enriched in the IL12 signaling pathway, and up-regulated expression of IL12RB2 in rosacea lesions. The expression of IL12RB2 is up-regulated by IFN-γ in Th1 cells and plays a role in Th1 cell differentiation *via* leading to the formation of high-affinity IL12 binding sites and reconstitution of IL12 dependent signaling (26). Overall, distinct transcriptional profiles are linked to inflammation at lineage-specific transcriptional regulators in rosacea lesions. Taken together, we suggest that IFNγ/STAT1 is the core of the critical gene regulatory network connecting the epithelial–immune crosstalk in rosacea development, and targeting IFNγ/STAT1 might be a potential therapy for the treatment of rosacea.

### Epidermal-Derived IFNγ/STAT1/IRF1 Signature Contributes to Rosacea Lesions

As immune sentinels, keratinocytes produce innate immune mediators and act as non-professional antigen-presenting cells (27). Exposure to acute solar ultraviolet (SUV) leads to upregulation of IFNγ and downstream STAT1/IRF1 signaling in the epidermis (22). These findings suggest that the IFNγ/STAT1/IRF1 signature is derived from epidermis in rosacea. According to our epidermal RNA-seq data, the upregulated genes were mainly related to the IFNγ signaling pathway for rosacea lesions. Furthermore, *STAT1 and IRF1* were identified across all rosacea subtypes. Our findings, together with previous reports, support that epidermis is pivotal for immune cell activation, which in turn leads to amplification of local innate immune responses. Similar “transcription circuits” including STAT1/IRF1 were found in psoriatic epidermis (28). Our findings provide a new model that diverse inflammatory processes in rosacea may be driven largely by just a small number of hubs within the epidermal transcription circuitry, such as STAT1/IRF1.

Given the importance of epithelial–immune crosstalk, there is value in measuring the expression from immune cells in rosacea lesions. Deconvolution is an accurate method for detecting a signal that is specific for a cell type and is expressed in a consistent pattern among the cell types of a heterogeneous sample (14). Resting dendritic cells and macrophages were common in ETR, PPR and PHR subtypes, which highlight the participation of innate immunity in rosacea pathogenesis. Furthermore, in terms of the effect of STAT1/IRF1 on M1 polarization, our previous study found that ADAMDEC1 plays a pro-inflammatory role in rosacea by modulating the M1 polarization of macrophages (29). Macrophages and keratinocytes influence the expression of inflammatory modulators that manifest clinically in the formation of pustules, papules, and sensations of heat and pain (30). The dialogue between keratinocytes and immune cells is fine-tuned to facilitate coordinated responses to preserve homeostasis and mount a host defense (7). Our data suggest that over-active keratinocyte–macrophage crosstalk might be an argument for activation of the innate immune system for rosacea development.

### Subtype-Specific Gene Signatures Correlated With Clinical Phenotypes

Various subtypes can be manifested in one patient, thus leading to overlapping or combined clinical manifestations. To gain exact molecular insight into rosacea subtypes, we collected typical erythema, papules and phymatous lesions from ETR, PPR and PHR subtypes and compared the rosacea subtypes with healthy controls. We found that cornification and formation of the cornified envelope in ETR and PPR indicated epidermal dysfunction (skin barrier) as an early phase of rosacea. Previous study of the PPR subtype found it characterized by a profoundly diminished skin barrier (18); our results further support the prominent permeability barrier alterations in ETR at the molecular level, which highlight the diminished skin barrier as a potential initiator of rosacea.

We further explored the subtype-specific characteristics by comparing one subtype with the other two. The ETR subtype featured striated muscle contraction, which is consistent with the clinical symptoms of frequent episodes of transient facial erythema and/or persistent erythema. The immunoregulatory interaction was predominantly identified in the PPR subtype, which indicates an inflammatory infiltrate leading to papules and pustules. Compared with the PPR subtype, the down-regulated genes enriched in the GO terms of keratinization, neutrophil chemotaxis, monocyte chemotaxis and extracellular matrix disassembly in PHR subtype. The down-regulated genes involved in monocyte chemotaxis including CCL4 and CCL5, indicated the macrophage activation from M1 to M2 type (31), which plays critical roles in tissue remodeling and repair. Our data is consistent with the previous study (32), which indicated non-phagocytic macrophages favors a role in limiting fibrosis. PHR also exhibited upregulation of many genes involved in lipid metabolism, thus indicating sebaceous gland hyperplasia with a strong connection between chronic inflammatory processes and skin fibrosis development. Our work provides a detailed characterization of transcriptomic diversity of rosacea subtypes and additional evidence that this can be linked to phenotypes, from epidermis (ETR and PPR) to sebaceous gland (PHR), vascular reaction (ETR) and cell infiltration (PPR) of acute inflammation to hyperplasia (PHR) of chronic inflammation.

Among the immune cell types, the transcriptome composition of mast cells was particularly high in the ETR subtype, which suggests that mast cells participate in the initial phase of rosacea evolution. Mast cell proteases recruit immune cells to amplify the inflammatory response (cathelicidin LL-37 activation) and cause vasodilation (33). For inflammatory outbreaks in the PPR, CD4^+^ and CD8^+^ T cells and B cells were predominant in PPR, which supports the cell-mediated immune response. As compared with ETR and PPR, PHR shows absence of distinct immune cell composition. Here our novel immune-cell composition analysis provided new insights into the abundance of immune cell types of rosacea subtypes from patients with early erythema to fibrotic changes.

In conclusion, we extensively and comprehensively investigated the characteristics of central facial skin and rosacea lesions in a stepwise manner. We potentially revealed the communication between the skin barrier and innate immune system, keratinocyte–immune crosstalk, and provided insights into the initial keratinocyte pattern for the pathogenesis of rosacea. Finally, our findings suggested STAT1 might be a potential therapeutic target for rosacea.

## Data Availability Statement 

All data needed to evaluate the conclusions in the study are provided in the manuscript and/or the Supplementary Materials, further inquiries can be directed to the corresponding author/s. Whole-skin and epidermal sequencing data from rosacea patients have been deposited in the genome sequence archive under accession number HRA000379 and HRA000809 (http://bigd.big.ac.cn/gsa-human/browse).

## Ethics Statement

The studies involving human participants were reviewed and approved by the ethics committee of Xiangya Hospital, Central South University. The patients/participants provided their written informed consent to participate in this study.

## Author Contributions

ZD and FL performed most of the experiments, analyzed the data and wrote the manuscript. MC, WX, CH, SG, DJ, YO, SX, JML, and QS collected the clinical samples. HX provided technical support and suggestions for the project. JL, GZ, and ZD conceived the project and supervised the study. GZ, ZD and JL designed the experiments, analyzed and interpreted data, and wrote the manuscript. All authors contributed to the article and approved the submitted version.

## Funding

This work was supported by the National Natural Science Foundation of China (No. 81874251, No. 81673086, No. 81773351, No. 82073457), and by the Science and Technology Innovation Plan of Hunan province (No. 2018SK2087) and Science and Technology Aid Program of Xinjiang Uygur Autonomous Region (No. 2019E0289).

## Conflict of Interest

The authors declare that the research was conducted in the absence of any commercial or financial relationships that could be construed as a potential conflict of interest.
