# Supplementary material for: Keratinocyte-Immune Cell Crosstalk in a STAT1-Mediated Pathway: Novel Insights Into Rosacea Pathogenesis
Source: Front Immunol. 2021 Jul 5;12:674871. doi: 10.3389/fimmu.2021.674871 (PMC8287635; doi:10.3389/fimmu.2021.674871)
Supplement: Supplementary file 1 [file DataSheet_1.pdf]

Supplementary figures and figure legends

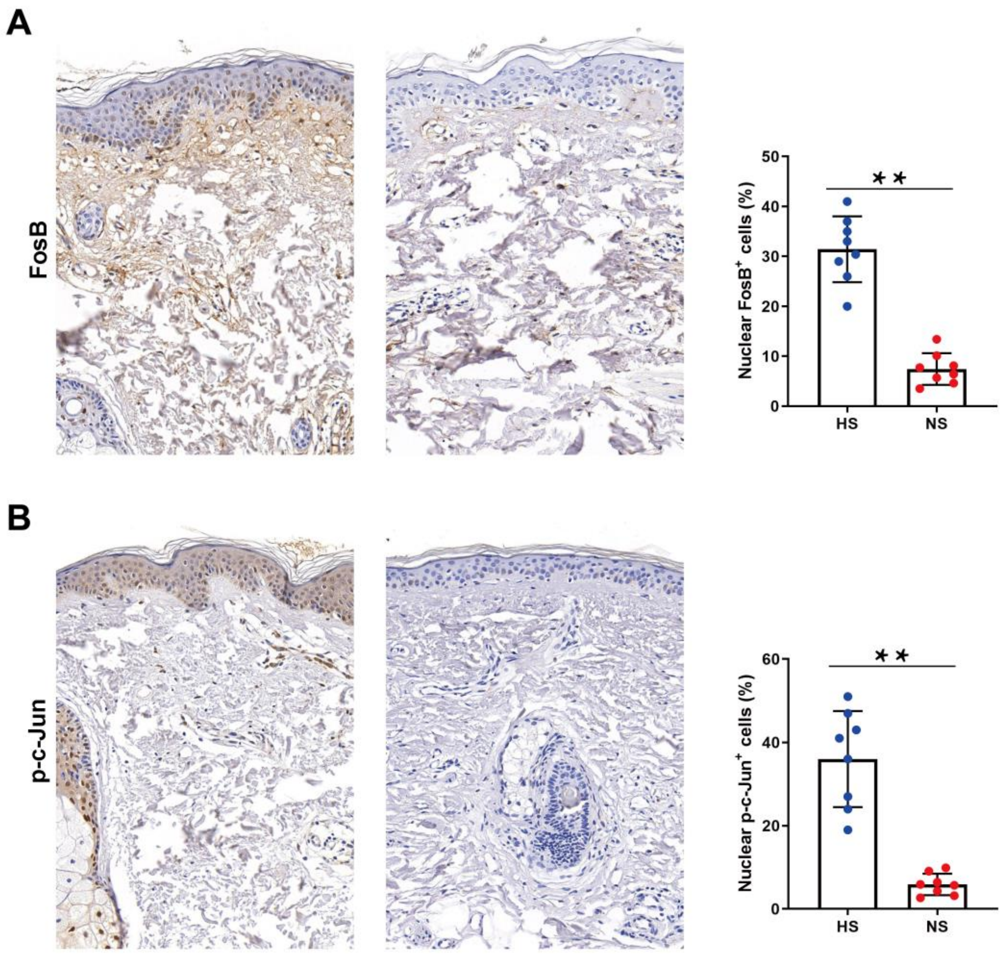

Supplementary Figure S1. Transcription factors (TFs) related to transcriptional profiling in central facial skin. Immunohistochemistry staining for validation of FosB and p-c-Jun. HS, healthy skin (n = 8). NS, normal skin surrounding the auricle in rosacea patients (n = 8). Right panel, the quantification of nuclear localization of FosB and p-c-Jun. Data represent the mean ± SEM. **P < 0.01. 2-tailed unpaired Student's t-test was used.

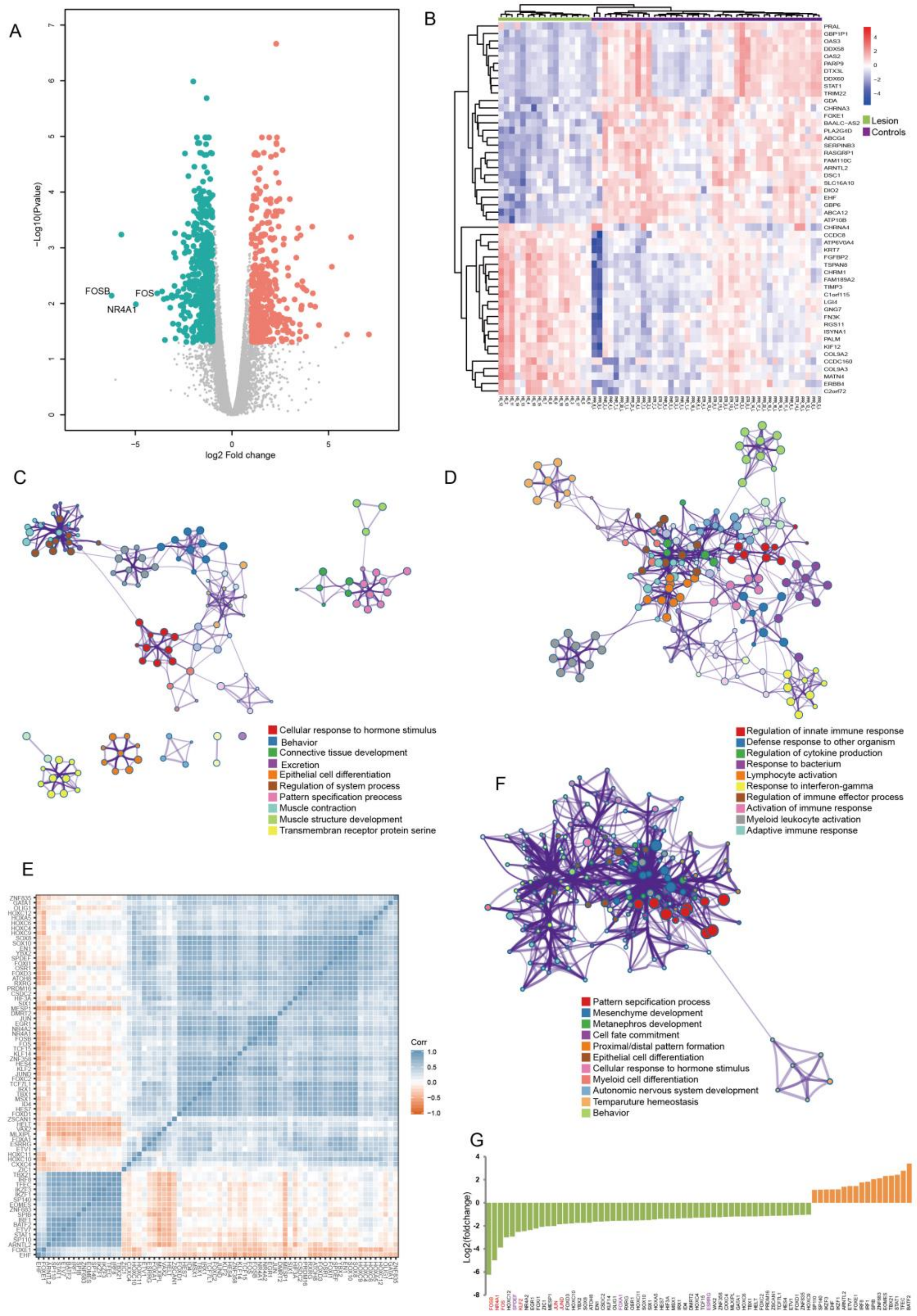

Supplementary Figure S2. Transcriptional profiling in rosacea lesions as compared with central facial skins from health volunteers

A. Volcano plot for the differentially expressed genes (DEGs) in rosacea lesions (LS, N=42) compared with central facial skins (HS N=17) from

healthy volunteers. The upregulated genes in rosacea lesions are in red and downregulated genes are in green. Genes without significant diversity are in gray.

B. Heatmap generated using the top 50 DEGs in comparing LS vs HS.
C. Metascape network analysis of intra-cluster redundancies and inter-cluster similarities of enriched GO terms and pathways for downregulated genes.
D. Metascape network analysis of enriched GO terms and pathways for upregulated genes.
E. Correlation heatmap of significant correlation for expression of all 72 transcription factors.
F. Metascape network analysis of enriched GO terms and pathways for transcription factors.
G. Log2 fold change values for the different expression of all 72 transcription factors with downregulated expression of AP1 TFs (FOS, FOSB, JUN, and JUND).

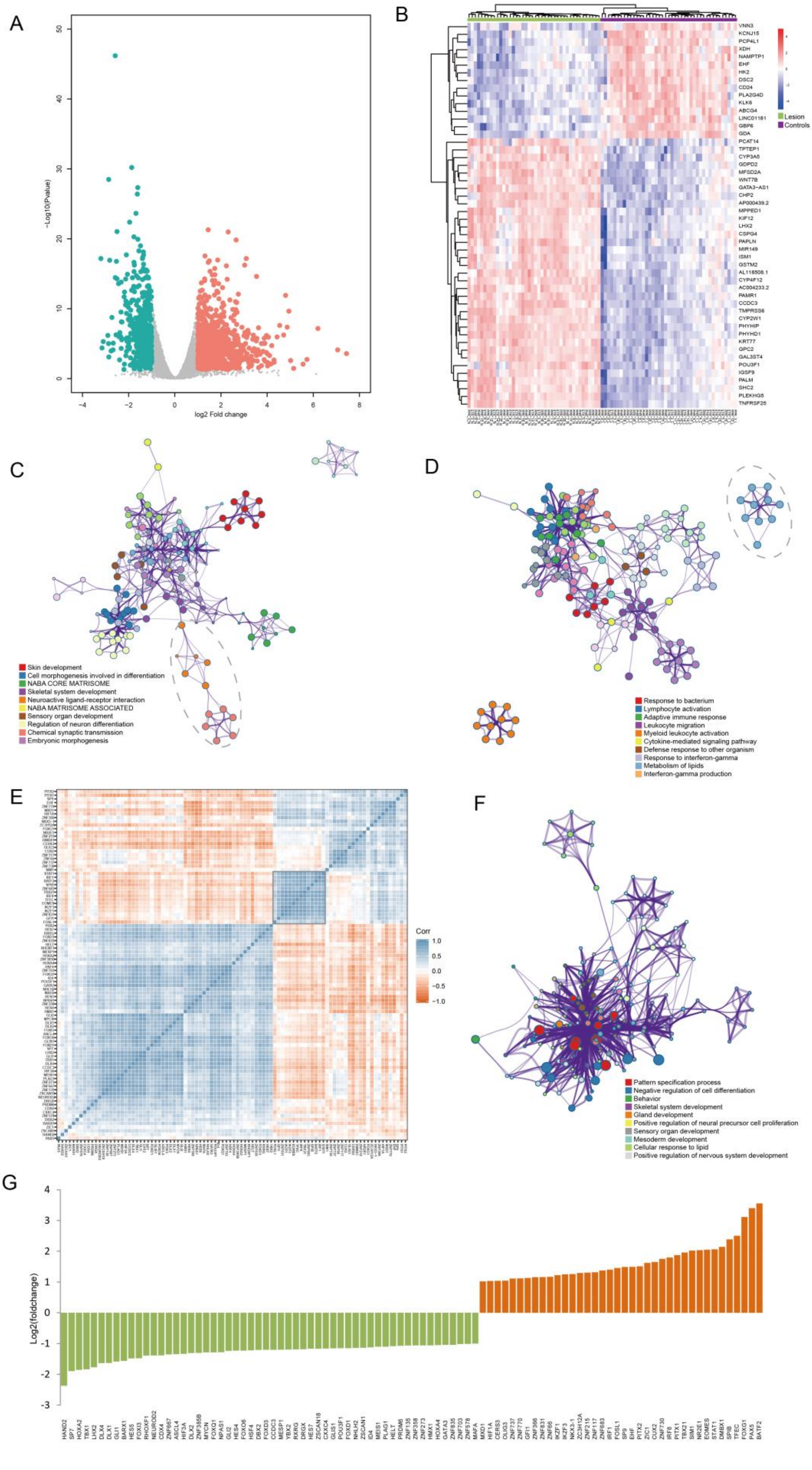
A
−Log10(Pvalue)
log2 Fold change
B
VNN3
KCNJ15
PCP4L1
XDH
NAMPTP1
EHF
HK2
DSC2
CD24
PLA2G4D
KLK6
ABCG4
LINC01181
GBP6
GDA
PCAT14
TPTEP1
CYP3A5
GDPD2
MFSD2A
WNT7B
GATA3−AS1
CHP2
AP000439.2
MPPED1
KIF12
LHX2
CSPG4
PAPLN
MIR149
ISM1
GSTM2
AL118508.1
CYP4F12
AC004233.2
PAMR1
CCDC3
TMPRSS6
CYP2W1
PHYHIP
PHYHD1
KRT77
GPC2
GAL3ST4
POU3F1
IGSF9
PALM
SHC2
PLEKHG5
TNFRSF25
Lesion
Controls
C
Skin development
Cell morphogenesis involved in differentiation
NABA CORE MATRISOME
Skeletal system development
Neuroactive ligand-receptor interaction
NABA MATRISOME ASSOCIATED
Sensory organ development
Regulation of neuron differentiation
Chemical synaptic transmission
Embryonic morphogenesis
D
Response to bacterium
Lymphocyte activation
Adaptive immune response
Leukocyte migration
Myeloid leukocyte activation
Cytokine-mediated signaling pathway
Defense response to other organism
Response to interferon-gamma
Metabolism of lipids
Interferon-gamma production
E
Corr
1.0
0.5
0.0
−0.5
−1.0
F
Pattern specification process
Negative regulation of cell differentiation
Behavior
Skeletal system development
Gland development
Positive regulation of neural precursor cell proliferation
Sensory organ development
Mesoderm development
Cellular response to lipid
Positive regulation of nervous system development
G
Log2(foldchange)
HAND2
SP7
HOXA2
TBX1
LHX2
DLX4
DLX1
GLI1
BARX1
HES5
FOXI3
RHOXF1
NEUROD2
CDX4
ZNF667
ASCL4
HIF3A
DLX2
ZNF385B
MYCN
FOXQ1
NFIA51
GLI2
HES4
FOXO6
HSF4
DBX2
FOXD3
CCDC3
MESP1
YBX2
RXRG
DRGX
HES7
ZSCAN18
CXXC4
GLIS1
POU3F1
FOXD1
NHLH2
ZSCAN1
ID4
MEIS1
PLAG1
HELT
PRDM6
ZNF135
ZNF358
ZNF273
HMX1
HOXA4
GATA3
ZNF835
ZNF703
ZNF578
MAFA
MXD1
HIF1A
CERS3
CUX3
ZNF737
GFI1
ZNF770
ZNF366
ZNF831
ZNF66
IKZF1
IKZF3
NKX3-1
ZC3H12A
ZNF215
ZNF117
ZNF683
IRF1
FOSL1
SP9
EHF
PITX2
ZIC1
CUX2
ZNF730
IRF8
PITX1
TBX21
SIM1
NR2E1
EOMES
STAT1
DMBX1
SPIB
TFEC
FOXG1
PAX5
BATF2

Supplementary Figure S3. Transcriptional profiling in rosacea lesions as compared with matched skin surrounding the auricle

A. Volcano plot for the DEGs between rosacea lesions (LS) and matched skin surrounding the auricle (NS, N=42).

B. Heatmap of the top 50 DEGs between rosacea lesions (purple color bar) and matched skin surrounding the auricles (green color bar).

C. Metascape network analysis of GO terms and pathways enriched for downregulated genes, indicating genes involved in biological processes of skin development, neuroactive ligand-receptor interaction and chemical synaptic transmission.

D. Metascape network analysis of the GO terms and pathways enriched for upregulated genes, mainly involved in biological processes of response to bacterium and metabolism of lipids.

E. Correlation heatmap of significant correlation for expression of all 94 transcription factors.

F. Metascape network analysis of transcription factors highlighting the pattern specification process.

G. Log2 fold change values for the different expression of all 94 transcription factors.

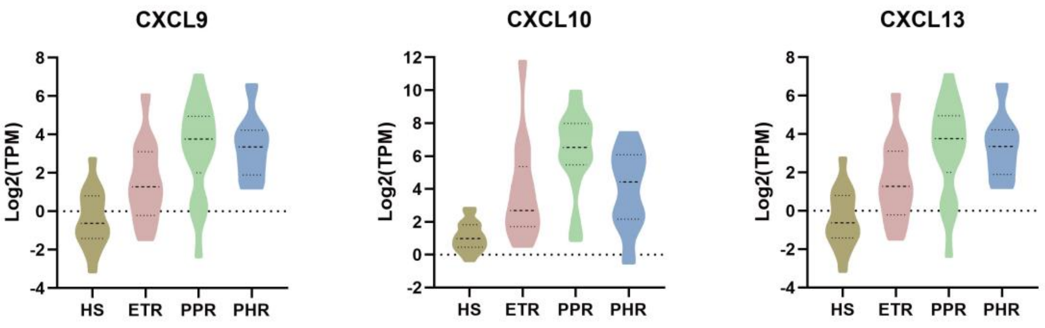

Supplementary Figure S4. Violin plots showing CXCL9, CXCL10 and CXCL13 expression in rosacea lesions (LS, N=42) compared with central facial skins (HS N=17) from healthy volunteers.

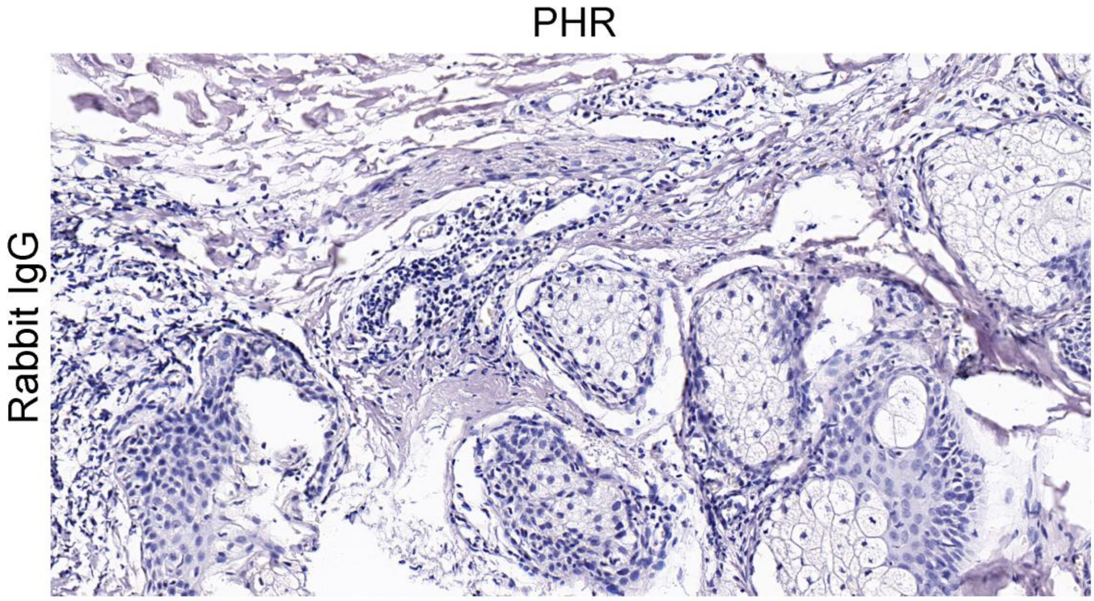

Supplementary Figure S5. Immunohistochemistry staining for rabbit IgG in PHR skin lesion as the negative control.
